# Contrasting seasonal responses in dinitrogen fixation between shallow and deep-water colonies of the model coral *Stylophora pistillata* in the northern Red Sea

**DOI:** 10.1371/journal.pone.0199022

**Published:** 2018-06-14

**Authors:** Vanessa N. Bednarz, Malik S. Naumann, Ulisse Cardini, Nanne van Hoytema, Laura Rix, Mamoon M. D. Al-Rshaidat, Christian Wild

**Affiliations:** 1 Coral Reef Ecology Group, Leibniz Centre for Tropical Marine Research (ZMT), Bremen, Germany; 2 Marine Department, Centre Scientifique de Monaco, Principality of Monaco; 3 Integrative Marine Ecology Department, Stazione Zoologica Anton Dohrn, Villa Comunale, Naples, Italy; 4 RD3 Marine Microbiology, GEOMAR Helmholtz Centre for Ocean Research, Kiel, Germany; 5 Laboratory for Molecular Microbial Ecology, Marine Science Station, Aqaba, Jordan; 6 Molecular and Microbial Ecology, Department of Biological Sciences, The University of Jordan, Amman, Jordan; 7 Marine Ecology Group, Faculty of Biology and Chemistry, University of Bremen, Bremen, Germany; Academia Sinica, TAIWAN

## Abstract

Tropical corals are often associated with dinitrogen (N_2_)-fixing bacteria (diazotrophs), and seasonal changes in key environmental parameters, such as dissolved inorganic nitrogen (DIN) availability and seawater temperature, are known to affect N_2_ fixation in coral-microbial holobionts. Despite, then, such potential for seasonal and depth-related changes in N_2_ fixation in reef corals, such variation has not yet been investigated. Therefore, this study quantified seasonal (winter vs. summer) N_2_ fixation rates associated with the reef-building coral *Stylophora pistillata* collected from depths of 5, 10 and 20 m in the northern Gulf of Aqaba (Red Sea). Findings revealed that corals from all depths exhibited the highest N_2_ fixation rates during the oligotrophic summer season, when up to 11% of their photo-metabolic nitrogen demand (CPND) could be met by N_2_ fixation. While N_2_ fixation remained seasonally stable for deep corals (20 m), it significantly decreased for the shallow corals (5 and 10 m) during the DIN-enriched winter season, accounting for less than 2% of the corals’ CPND. This contrasting seasonal response in N_2_ fixation across corals of different depths could be driven by 1) release rates of coral-derived organic matter, 2) the community composition of the associated diazotrophs, and/or 3) nutrient acquisition by the *Symbiodinium* community.

## Introduction

Scleractinian corals are effectively composed of an assemblage of diverse organisms (often referred to as the coral ‘holobiont’) including the cnidarian host, endosymbiotic dinoflagellates (of the genus *Symbiodinium*), bacteria, archaea and fungi [1]. *Symbiodinium* provides the heterotrophic coral host with carbon (C)-rich photosynthates that are essential for host survival in oligotrophic reef environments, where access to heterotrophic C sources is often limited [2]. However, net coral growth also requires a sufficient supply of nitrogen (N), another limiting nutrient in tropical reefs waters [3]. In order to cope with the limited N availability, corals can acquire dissolved inorganic nitrogen (DIN) from surrounding seawater (even at very low concentrations) and have evolved efficient internal N cycling between the coral host and its photosynthetic symbionts [4–6]. In addition, corals are associated with dinitrogen (N_2_)-fixing bacteria (diazotrophs) that are able to convert dissolved elemental N_2_ into ammonium via nitrogenase activity [7,8]. Thus, diazotrophs may compensate for the limited DIN availability in oligotrophic reef waters by providing an additional source of N that can be assimilated and metabolized by the coral host [7,9–12].

Corals harbor both autotrophic and heterotrophic diazotrophs whose N_2_ fixation activity largely depends on the prevailing environmental conditions [13]. Elevated temperature stimulates N_2_ fixation in corals [14], likely by increasing the enzymatic activity of nitrogenase [15]. Conversely, high environmental DIN concentrations can decrease N_2_ fixation, as the process is metabolically costlier for diazotrophs than DIN assimilation [16]. Another key factor regulating N_2_ fixation, particularly in autotrophic diazotrophs, is ambient light availability [17]. Although autotrophic diazotrophs require light for photosynthesis, high levels of photosynthesis-derived oxygen (O_2_) can inhibit the O_2_-sensitive nitrogenase enzymes [18]. On relatively high-latitude coral reefs—such as those of the northern Red Sea (e.g. 29°N for reefs in Jordan’s Gulf of Aqaba)—temperature, DIN concentrations and light availability differ significantly across seasons [19,20]. Previous studies on coral-associated diazotrophs in the northern Red Sea report highest N_2_ fixation rates during summer, when light levels and temperature are highest and DIN concentrations are lowest [12,21]. Cardini et al. (2015) concluded that diazotrophically-derived N sustains the high primary productivity of corals during nutrient-depleted summer conditions (<0.1 μM DIN) by contributing up to 11% of the corals’ photo-metabolic nitrogen demand (CPND), as opposed to only 2% during winter.

However, key abiotic parameters do not only change over temporal scales, but also over spatial scales such as along bathymetric and depth gradients [3]. On tropical coral reefs, light penetration decreases most rapidly to ~20 m, while temperature and inorganic nutrient concentrations stay constant within this depth range [22,23]. Corals undergo several adaptations in response to reduced light attenuation, such as morphological changes to optimize light harvesting [24], a shifting reliance from autotrophic to heterotrophic food sources [25,26] and changes in the associated *Symbiodinium* community [26,27]. The coral-associated diazotrophic community also undergoes changes along bathymetric gradients, with differences already apparent between 5 and 15 m depth [28,29]. Since diazotroph assemblages can differ across depths, the overall N_2_ fixation activity associated with these coral holobionts is also hypothesized to vary across depths. In addition, diazotroph assemblages located at different depths could also be hypothesized to demonstrate variable N_2_ fixation rates across seasons, especially given the aforementioned temporal changes in DIN levels. A recent study using the ^15^N_2_ tracer technique compared net assimilation rates of fixed N_2_ in shallow (5 m) and mesophotic (50 m) specimens of the scleractinian coral *Stylophora pistillata*, and the authors observed higher rates in the latter [11]. This difference was linked to an increased dependence on heterotrophy in mesophotic corals, however the choice of comparing shallow and mesophotic corals with clearly contrasting auto- vs. heterotrophic strategies may have masked the primary effect of depth-mediated light availability. Furthermore, the authors quantified depth-specific N_2_ fixation rates in these corals only during one season, whereas a depth-specific seasonal response has not been investigated yet. In order to tease apart the effects of light and other seasonal factors in conspecifics with hypothetically similar nutritional strategies, we investigated N_2_ fixation by the scleractinian coral *S*. *pistillata* along a shallower depth gradient (5−20 m) during two seasons (winter and summer) in the northern Gulf of Aqaba (Red Sea). Coral-associated N_2_ fixation rates were quantified using the acetylene reduction assay in laboratory incubation experiments. In addition, gross photosynthesis rates (*P*_g_) were measured in order to examine the respective autotrophic-heterotrophic status of the corals and to quantify the contribution of N_2_ fixation to the corals’ photo-metabolic N demand (CPND). We hypothesized similar seasonal responses in corals from all depths, with highest N_2_ fixation rates during summer due to the lower environmental DIN concentrations during this season.

## Materials and methods

### Coral collection and maintenance

**Fig 1:**
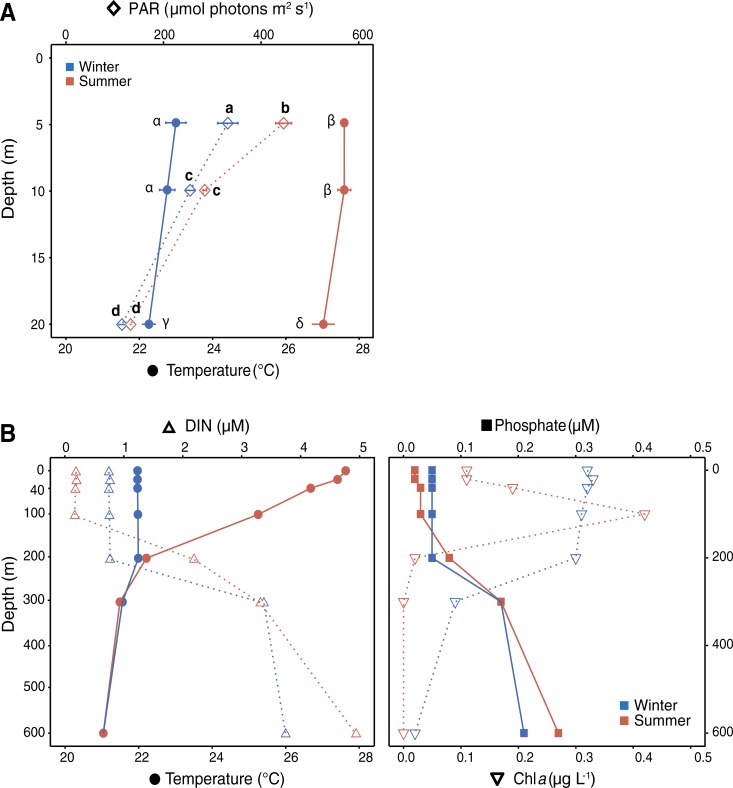
Environmental conditions at the study site. Environmental parameters (mean ± SD) measured at 5, 10 and 20 m depth at the study site (A) and along a 0–600 m depth gradient in the water column in the Gulf of Aqaba (B) during March 2013 (winter) and September 2013 (summer). Different lettering in panel A indicates significant differences for light levels (a-d) and water temperature (α-δ) between depths and seasons based on two-factor permutational ANOVAs with pairwise and Bonferroni corrected Monte Carlo tests (significance level, *p* < 0.05).

This study was conducted at a fringing coral reef located within a marine reserve in front of the Marine Science Station (MSS) at the northern Gulf of Aqaba (Red Sea), Jordan (29°27`N, 34°58`E). Permission for work within the marine reserve was issued by the University of Jordan and the MSS Aqaba. The fringing reef can be divided into a reef flat and a fore reef. Here, we focused on the fore reef, which consists of upper (4–8 m depth), middle (8–15 m depth) and lower (15–40 m depth) depth zones, each of which being characterized by distinctive 1) live coral cover, 2) coral species composition and 3) morphological features [30,31]. Live hard coral cover in the upper, middle and lower zone were approximately 15, 35 and 60%, respectively during the study period, with *S*. *pistillata* being abundant in each zone [2]. *S*. *pistillata* specimens (n = 7–8) were collected from individual colonies during two environmentally contrasting seasons, winter (02/03/2013) and summer (14/09/2013), by carefully chiseling fragments of similar size (5–6 cm in height, 1–2 cm diameter), morphology and pigmentation from the fore reef at 5, 10 and 20 m depth. To ensure biological replication as best as possible individual colonies were samples with a distance of at least 5 m in between. The distance between the individual sampling depth points along the gradual reef slope was approximately 50 m. Photosynthetically active radiation (PAR) and water temperature were measured seasonally at each depth using an underwater quantum sensor (LI-COR LI-192SA, Lincoln, Nebraska, USA) and HOBO loggers (Onset HOBO Pendant UA-002-64; temperature accuracy: ± 0.53°C, Bourne, MA, USA), respectively and averaged from daily measurements conducted over seven consecutive days (mean ± SD; Fig 1A). On these days, temperature was recorded over 24 h in 1 min intervals, while PAR was recorded during the daily maximum from 12:00 to 13:00 in 1 min intervals. Further environmental data (i.e. water temperature, nutrient and Chl *a* concentrations) were retrieved from the Israel National Monitoring Program (http://www.iui-eilat.ac.il/Research/NMPMeteoData.aspx), in order to demonstrate changes in environmental conditions along a wider bathymetric gradient (0 to 600 m depth). For this analysis, an open-water monitoring station close (*~* 6 km) to our study site was chosen, and data were compiled from the study period (March-September 2013; Fig 1B).

Coral specimens from each depth were individually glued with epoxy onto ceramic tiles and transferred to three inter-connected outdoor aquaria (800 L). Light intensities in the three aquaria were individually adjusted to comparative *in situ* light measurements at 5, 10 and 20 m depth, respectively, using variable layers of black mesh netting. Corals from each depth were placed in the aquarium with the depth-corresponding light intensity in order to avoid any change from *in situ* light levels. Adjusted light conditions (daily maximum) in the aquaria reached 350 and 450 μmol photons m^-2^ s^-1^ (5 m corals), 250 and 300 μmol photons m^-2^ s^-1^ (10 m corals) and 140 and 150 μmol photons m^-2^ s^-1^ (20 m corals) during winter and summer, respectively. The three aquaria were supplied with seawater freshly pumped from the reef at 10 m depth (exchange rate: 4000 L h^-1^) ensuring that water temperature (23.0°C in winter and 27.8°C in summer) and other environmental parameters (i.e. nutrients) were comparable between the light treatments. Corals were allowed to recover from fragmentation for 1 week before incubations were conducted in the aquaria under the depth-specific light conditions.

### Quantification of gross photosynthesis and N_2_ fixation rates

A detailed description of the chamber incubation procedure for quantifying *P*_g_ and coral-associated N_2_ fixation can be found in Bednarz et al. (2015). Briefly, net photosynthesis (*P*_n_) and respiration (*R*) rates were first quantified for all corals (n = 7–8 per depth and season) via O_2_ flux measurements over 90 min in the light (light intensities were 350, 250, 140 μmol photons m^-2^ s^-1^ during winter and 450, 300, 150 μmol photons m^-2^ s^-1^ during summer for 5, 10 and 20-m corals, respectively) and in the dark (at night) with a conductivity- and temperature-corrected O_2_ optode sensor (MultiLine^®^ IDS 3430, WTW GmbH, Weilheim, Bavaria, Germany, accuracy: ± 0.5% of measured value). All incubations were conducted in unfiltered seawater, and the O_2_ concentration in the incubation chambers changed by ± 10% after 90 min. This O_2_ difference was necessary to obtain measurable results without inducing stress in the corals [32]. O_2_ fluxes by the corals were calculated by subtracting the initial O_2_ concentrations from the final ones and correcting them with O_2_ fluxes measured in seawater control (without corals) incubations. Then, O_2_ fluxes were normalized to incubation time and the skeletal surface area of the corals. The skeletal surface area was measured using a standard geometric technique (Advanced Geometry) as described in [33]. Finally, *P*_g_ was calculated as *P*_g_ = *P*_n_*—R*. The total photosynthetic C acquisition (*P*_c_) was calculated from *P*_g_ by converting O_2_ fluxes into C equivalents based on molar weights and applying the formula *P*_c_ = μmol C produced x 12/PQ, assuming a 12 h daylight period and a photosynthetic quotient (PQ) equal to 1.1 previously determined for *S*. *pistillata* [34,35].

Following the O_2_ flux measurements, N_2_ fixation was measured on the same coral specimens using an adapted acetylene (C_2_H_2_) reduction technique [36,37]. Corals were transferred without aerial exposure into 1-L chambers filled with 0.8 L of seawater. Additional chambers only filled with seawater served as controls. Immediately prior to the start of the incubations, 10% of the seawater was replaced by freshly produced C_2_H_2_-saturated seawater. Chambers were then sealed ‘gastight’ before 10% of the headspace was replaced by freshly generated C_2_H_2_ gas. All chambers were positioned under the depth-specific light conditions and incubated under constant stirring (600 rpm) for a full dark-light cycle (24 h). Gas samples were drawn after 0 and 24 h and analyzed for ethylene (C_2_H_4_) concentrations using a customized reducing compound photometer (Peak Laboratories, Mountain View, CA, USA, detection limit = 100 ppb). C_2_H_4_ evolution in each coral incubation chamber was seawater control corrected and calculated according to [38]. Finally, N_2_ fixation rates were normalized to incubation time and the skeletal surface area of the corals. In order to estimate the CPND, measured acetylene reduction rates were converted into N equivalents using a conservative theoretical 4:1 (C_2_H_4_:N_2_) conversion ratio [36,39]. The photo-metabolic N demand was then calculated from the *P*_c_ rates assuming that only ~25% of the photosynthetically fixed C was incorporated into *Symbiodinium* and host biomass (with the remaining fixed C assumed to be respired and released as organic C to the surrounding seawater as previously determined for *S*. *pistillata* [40]) and assuming a C:N ratio of 7 [25]. Previously, a C:N ratio of 7 has been determined for *Symbiodinium* of *S*. *pistillata* corals collected from a 5 to 20 m depth gradient in the Gulf of Aqaba [25].

### Statistical analysis

Data were analyzed using non-parametric permutational analysis of variance (PERMANOVA) in a univariate approach, since assumptions (i.e. normal distribution) for parametric analyses were not met. Analyses were carried out using Primer-E version 6 software [41] with the PERMANOVA+ add on [42]. Two-factor PERMANOVAs were performed to test for differences in light availability, seawater temperature, N_2_ fixation, *P*_g_ and CPND between the three depths and the two seasons. Bray-Curtis similarities and type III (sequential) sum of squares were used for analyses with permutation of residuals under a reduced model (9999 permutations). The significance for the main test and for the pair-wise comparisons was based on Monte Carlo tests with Bonferroni corrected *p*-values to account for multiple comparisons (significance level, *p* < 0.05).

## Results and discussion

**Fig 2:**
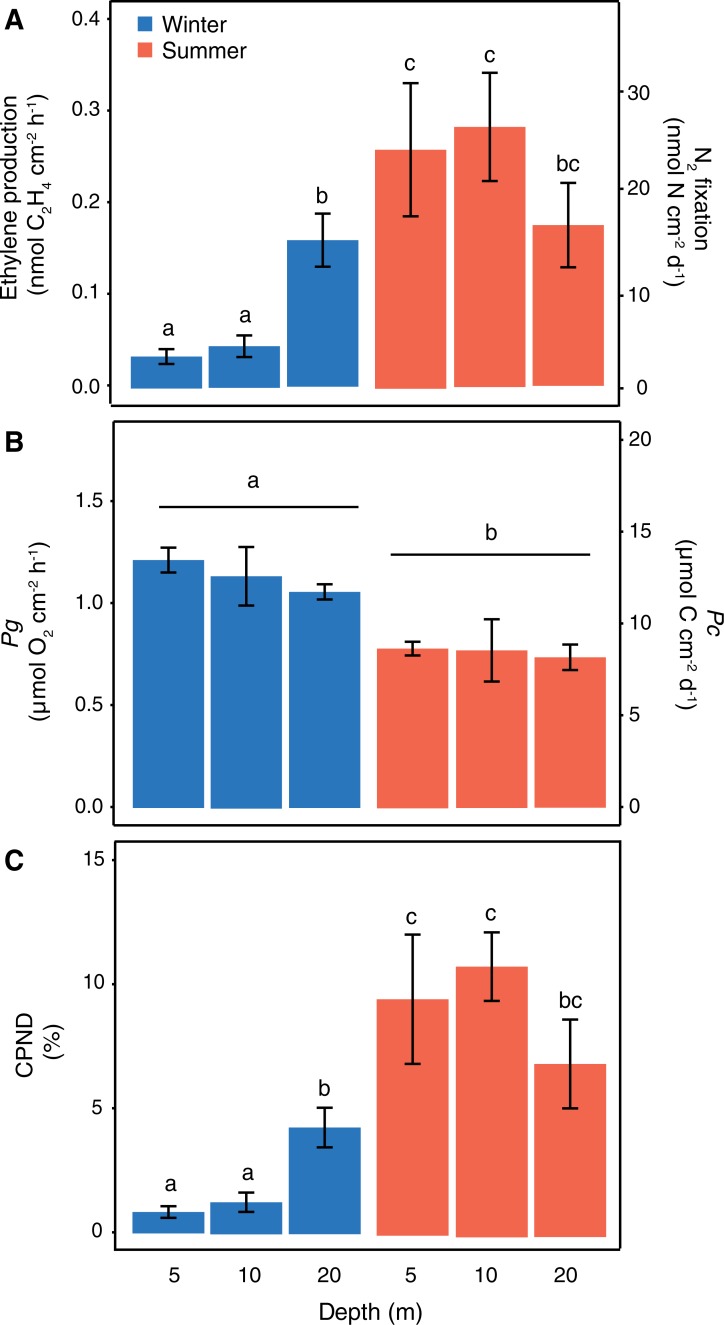
N_2_ fixation and photosynthesis rates in *Stylophora pistillata*. N_2_ fixation expressed as ethylene production or amount of nitrogen fixed (A), gross photosynthesis expressed as *P*_g_ or *P*_c_ (B) and the contribution of fixed nitrogen to the photo-metabolic nitrogen demand (C) in *Stylophora pistillata* corals. All rates were quantified in corals collected from three different depths (5, 10 and 20 m) during winter and summer (n = 7–8; mean ± SE). Different lettering (a-c) indicates significant differences between depths and seasons based on two-factor permutational ANOVAs with pairwise and Bonferroni corrected Monte Carlo tests (significance level, *p* < 0.05).

Previous studies have described either seasonal or depth-specific differences in coral-associated N_2_ fixation rates, while the present study provides a comparison of seasonal differences across corals from different depths. The investigated environmental parameters (i.e. seawater temperature and light availability) varied differently across seasons and depths. Differences in water temperature (and likely also nutrient availability) were most pronounced on a seasonal scale than across the investigated depth range, whereas light levels decreased significantly from 5 to 20 m depth and varied seasonally only at shallower depths (Fig 1A). Overall, corals from all investigated depths showed active N_2_ fixation in both seasons, as indicated by higher C_2_H_4_ concentrations measured in coral incubations compared to seawater controls. The acetylene reduction technique provides information about gross N_2_ fixation, rather than about the actual assimilation of fixed N_2_ by the coral. Here, we assume that ‘most’ of the N_2_ fixation-derived N was assimilated by the corals, since our N_2_ fixation rates (0.1–0.3 nmol C_2_H_4_ cm^-2^ h^-1^ or 3.4–27.3 nmol N cm^-2^ d^-1^; Fig 2A) are in the same range as previously reported for *S*. *pistillata* from the Gulf of Aqaba using the ^15^N_2_ tracer technique [11]. Similar N_2_ fixation rates in *S*. *pistillata* colonies were also reported from the Great Barrier Reef [29], while conspecifics from New Caledonia showed 10-times higher rates [43]. Besides measurement and technique-associated differences, such geographic variations may also suggest that certain locations are characterized by environmental conditions that stimulate the abundance and/or activity of coral-associated diazotrophs. However, it is still under debate whether diazotroph-derived N is actually translocated from the bacteria to the coral-algae symbiosis. Thus, localizing and tracing the fate of this N within different cells of the coral holobiont will be required to ultimately understand the role of diazotrophs in coral nutrition.

In the present study, N_2_ fixation was found to differ significantly across seasons, although this seasonal effect only occurred for corals from shallower (5 and 10 m) depths (Table 1 and Fig 2A). These corals from 5 and 10 m depths (hereafter referred to as “shallow corals”) were characterized by statistically significant, 6-fold higher rates of N_2_ fixation in summer as compared to winter. By contrast, corals from 20 m (hereafter referred to as “deep corals”) fixed N_2_ at similar rates in both seasons. *P*_g_ rates of corals from all depths were similar within each season (Fig 2B), demonstrating that the seasonal variability in N_2_ fixation rates is independent of the coral’s autotrophic status (at least in colonies of the depths surveyed).

**Table 1:** Statistical results for differences in environmental conditions and coral-associated physiological parameters between depths and seasons.

Variables	Effect	*df*	SS	MS	Pseudo *F*	*p* (MC)	Fig
PAR	Depth (De)	2	85.48	42.74	244.84	**<0.001**	1A
(μmol photons m^-2^ s^-1^)	Season (Se)	1	5.31	5.31	30.41	**<0.001**	
	De x Se	2	3.36	1.68	9.62	**<0.001**	
	Residuals	108	18.85	0.17			
	Total	113	113				
Seawater temperature	Depth (De)	2	1.59	0.79	60.18	**<0.001**	1A
(°C)	Season (Se)	1	109.94	109.94	8336.60	**<0.001**	
	De x Se	2	0.05	0.02	1.83	0.165	
	Residuals	108	1.42	0.01			
	Total	113	113				
N_2_ fixation (nmol C_2_H_4_ cm^-2^ h^-1^ or) nmol N cm^-2^ d^-1^)	Depth (De)	2	5514	2757	3.205	**0.011**	2A
	Season (Se)	1	17351	17351	20.171	**<0.001**	
	De x Se	2	9047	4524	5.2587	**<0.001**	
	Residuals	38	32688	860			
	Total	43	66121				
Gross photosynthesis (μmol O_2_ cm^-2^ h^-1^ or μmol C cm^-2^ d^-1^)	Depth (De)	2	392	196	1.176	0.318	2B
	Season (Se)	1	4199	4199	25.209	**<0.001**	
	De x Se	2	118	59	0.356	0.764	
	Residuals	38	6329	167			
	Total	43	11076				
CPND (%)	Depth (De)	2	7160	3580	3.893	**0.004**	2C
	Season (Se)	1	26171	26171	28.641	**<0.001**	
	De x Se	2	8445	4222	4.592	**<0.001**	
	Residuals	38	34943	920			
	Total	43	78386				

Results of the two-factorial permutational ANOVAs for testing the effects of depth (5, 10 and 20 m) and season (winter and summer) on photosynthetically active radiation **(**PAR) and seawater temperature during the study period as well as on N_2_ fixation, gross photosynthesis and on the contribution of N_2_ fixation to the corals’ photo-metabolic nitrogen demand (CPND) in *Stylophora pistillata*. Statistically significant Monte Carlo (MC) *p*-values (<0.05) are highlighted in bold.

The annual stratification cycle in the Gulf of Aqaba results in pronounced seasonal fluctuations in environmental parameters, such as water temperature and nutrient levels [19,44]. In summer the formation of a nutricline at ~100 m depth causes nutrient depletion in the stratified upper water column, while deep-water mixing during winter brings nutrient-rich seawater back into the reef zone (Fig 1B) [20,23]. Elevated DIN availability can inhibit the energy-costly process of N_2_ fixation in favor of DIN assimilation [45], whereas the more pronounced oligotrophic conditions in summer favor coral-associated N_2_ fixation [12,21]. Also, the abundance of potential diazotrophic bacteria associated with corals increases during seasons with reduced DIN availability in the seawater [46], indicating that corals might be able to acquire some additional N from these bacteria. In the present study, the CPND increased significantly from 1–4% during winter to 7–11% during summer (Fig 2C), suggesting that N_2_ fixation may compensate for the reduced DIN availability during summer by contributing more N to the N budget of *S*. *pistillata*. Since *S*. *pistillata* colonies of the Gulf of Aqaba normally experience decreased *Symbiodinium* densities, alongside increased Chl *a* content per *Symbiodinium* cell, during summer [12], N_2_ fixation-derived N may be relatively more important for coral productivity in this season; this fixed and presumable translocated N might also be important in re-establishing peak *Symbiodinium* densities at the end of the summer season [47,3]. Interestingly, only the shallow corals showed increased N_2_ fixation rates during summer, which is in line with previous seasonal observations on scleractinian and soft corals from 10 m depth in the Gulf of Aqaba [12,21]. In contrast, N_2_ fixation rates in deep corals were seasonally stable. Consequently, the CPND was seasonally stable in deep corals, while it significantly increased in shallow-water from winter to summer. This depth-specific seasonal response of coral-associated N_2_ fixation cannot be directly explained by the seasonally variable DIN availability, since DIN concentrations within each season were similar across the investigated depth range (5–20 m) (Fig 1). Rather, depth-driven light differences that alter coral physiology (e. g., mucus release by the coral host and nutrient acquisition by the in hospite *Symbiodinium* community) and/or variations in the diazotrophic community of the coral holobionts across depths may instead have affected N_2_ fixation rates.

The photosynthetic efficiency of *Symbiodinium* increases with light availability and correlates positively with the amount of C translocated to the coral host [48,49]. Thus, under higher light availability the coral host receives more C than required for its metabolism and releases the excess C as organic matter to the surrounding seawater [50]. Heterotrophic diazotrophs in particular depend on energy-rich organic matter that is assimilated from the surrounding seawater and/or provided by the coral host [51]. The coral mucus surface layer with its high organic C content [52,53] represents a suitable habitat for heterotrophic bacteria and contains high abundances of active diazotrophs [7,11,54]. Since depth- and seasonal-driven light differences change the quality and quantity of coral-derived mucus, they may consequently affect coral-associated N_2_ fixation. Indeed, total organic matter (i.e. mucus) release by shallow *S*. *pistillata* corals significantly increases during the summer season at the same study location [12], likely as a result of elevated light availability [55,56]. This would provide heterotrophic diazotrophs with an energy-rich food source and may explain the observed increased N_2_ fixation activity in shallow corals during summer. In contrast to their shallow-water conspecifics, deep-water corals are likely to release organic matter at consistent rates throughout the year due to seasonally less variable light availabilities, and this may account for the seasonally stable N_2_ fixation rates observed herein.

Besides light- and photosynthesis-driven changes in coral mucus release rates, the coral-associated diazotrophic communities themselves can also change along depth gradients, and this may have also contributed to the depth-related variation in N_2_ fixation rates. A recent study found significant differences in the diazotrophic community of *S*. *pistillata* colonies collected from 5 and 15 m depths on the Great Barrier Reef [29]. Interestingly, variations in light exposure significantly changed the community associated with 5 m corals, while no light effect was found for the community associated with 15 m corals. Since the diazotrophic community composition of shallow corals seems to show a more pronounced response to changes in light levels [29], light may also have a stronger effect on the activity of these bacteria. In the present study, shallow corals experienced a more pronounced seasonal change in light availability compared to the deep-water corals; such light level variation may have been associated with greater seasonal changes in the diazotrophic community, as well as diazotroph cell densities, and, therefore, resulted in the seasonally more variable N_2_ fixation rates in these shallow-water corals.

Besides changes in the diazotrophic community, the dominant coral-associated *Symbiodinium* genotype can also vary along depth gradients with certain *Symbiodinium* types (clades) being more efficient at photosynthesizing and assimilating nutrients than others *in hospite* [48,57,58]. This can lead to differing levels of photosynthate and nutrient transfer to the coral host [48,57,58] and may subsequently influence the coral’s response to seasonally changing environmental conditions (e.g. DIN availability). In the northern Red Sea, *S*. *pistillata* corals shift from hosting *Symbiodinium* clade A in shallow depths (5–10 m) to clade C below 40 m depth [24,27]. At intermediate depths of 20 m, *S*. *pistillata* starts to primarily host clade C over clade A [59]. The DIN assimilation capacity of clade A is ~10-times higher than for clade C, suggesting that shallow corals are able to utilize the increased DIN available during winter more efficiently than corals hosting clade C [58,60]. Consequently, shallow corals are likely to be less dependent on diazotrophically-derived N during winter, which may cause the significant drop in N_2_ fixation rates. Although speculative at this time, a physiological linkage between *Symbiodinium* genotype and N_2_ fixation may exist, since *Symbiodinium* can host their own diazotrophic community [61] and are the primary site for diazotrophically-derived N uptake within the coral symbiosis [28,43]. In future experiments, we recommend using corals experimentally infected with different *Symbiodinium* clades, such that the specific effect of host and *Symbiodinium* genotype on N_2_ fixation can be tested.

In conclusion, the results presented in this study indicate that, rather than a gradual change in coral-associated N_2_ fixation along the depth gradient, there is instead a division into two vertically distributed groups: 1) seasonally variable N_2_ fixation in shallow-water corals (0–15 m depth) and 2) seasonally stable N_2_ fixation in deep-water corals (20 m depth). In future experiments, it will be interesting to determine if the 1) activity, 2) abundance and 3) community composition of coral-associated diazotrophs also show a depth-specific response to globally changing environmental conditions, as well as whether any corresponding differences in N_2_ fixation activity have the potential to differentially influence the resilience and/or stress response of coral holobionts to climate change.
